# Investigating the Effect of Hydrogen Bonding on the Viscosity of an Aqueous Methanol Solution Using Raman Spectroscopy

**DOI:** 10.3390/molecules30153204

**Published:** 2025-07-30

**Authors:** Nan-Nan Wu, Fang Liu, Zonghang Li, Ziyun Qiu, Xiaofan Li, Junhui Huang, Bohan Li, Junxi Qiu, Shun-Li Ouyang

**Affiliations:** 1 School of Arts and Sciences, Guangzhou Maritime University, Guangzhou 510725, China; 2 School of Electrical Engineering, Shandong Huayu University of Technology, Dezhou 253000, China; lf13546566078@163.com; 3 School of Shipping and Maritime Studies, Guangzhou Maritime University, Guangzhou 510725, China; 17686215263@163.com (Z.L.); 17819317509@163.com (Z.Q.); xiaofanelvali@163.com (X.L.); hjh20050222@163.com (J.H.); 13054226527@163.com (B.L.); 17512900436@163.com (J.Q.)

**Keywords:** Raman spectroscopy, hydrogen bonding, methanol, viscosity

## Abstract

Water science has always been a central part of modern scientific research. In this study, the viscosity and hydrogen bond structures of methanol aqueous solutions with different molar ratios were investigated via confocal microscopic Raman spectroscopy. The Raman spectra of methanol in the CH and CO stretching regions were measured in order to investigate the structure of water/methanol molecules. The points of transition were identified by observing changes in viscosity following changes in concentration, and the bands were assigned to the C-H bond vibration shifts where the molar ratios of methanol and water were 1:3 and 3:1. Furthermore, the large band shift of 19 cm^−1^ between the methanol solutions with the lowest and highest concentrations contained three hydrogen bond network modes, affecting the viscosity of the solution. This study provides an explanation for the relationship between the microstructures and macroscopic properties of aqueous solutions.

## 1. Introduction

Water and methanol are the most commonly used solvents in natural sciences, engineering technology, agriculture, medicine, and economics, demonstrating their importance [1,2,3]. However, there is some controversy regarding their structures because of the complex hydrogen bonding networks involved [4,5,6]. The obvious importance of water as a solvation medium has fueled tremendous efforts to understand the properties of aqueous solutions. The anomalous properties of water are determined by continuous 3D networks of hydrogen bonds between neighboring molecules [7,8,9,10,11,12,13,14]. The variety of local environments of different OH groups leads to variations in the geometry and energy of their H-bonds. Alcohol molecules are typical organic molecules that contain hydrophobic groups and hydrophilic groups. Monohydric alcohols possess the simplest structures, making them ideal for studying hydrogen bonding in aqueous methanol solutions.

Raman spectroscopy is useful for studying structures in water [15,16,17,18,19] and has been used extensively to study the structures of methanol and its aqueous solutions [20,21] as it can effectively avoid the destruction of molecular structures in methanol solutions. Raman spectroscopy is the most sensitive method used for studying H-bonding in the stretching vibration regions of OH groups. Methanol molecules are believed to affect the hydrogen bonding networks of water, since methanol is an associating liquid that can both donate and accept hydrogen bonds, yet water–methanol mixtures also possess an OH group. Wavenumber shifts in Raman bands include a change in the microstructure of the solution and the formation of hydrogen bonds between methanol and water molecules. In order to understand the structural changes in methanol and methanol–water solutions, Ebukuro et al. [21] measured the frequency shifts in the Raman characteristic bands of methanol in both pure methanol and a methanol–water mixture at high temperature and high pressure. The results of their ab initio theory and Raman spectroscopy accounted for the spectral features of the two components in the binary mixture. Liu Huaibo et al. [22] used density functional theory to analyze the characteristic Raman bands of monohydric alcohols. Ouyang et al. [23] assigned characteristic Raman bands to different vibrational modes using a simulated Raman spectra calculation of methanol molecules. Many researchers [24,25,26,27,28,29,30,31] have investigated the physicochemical behavior of different concentrations of methanol–water mixtures with experimental DFT characterization to derive an optimized framework for methanol molecules in water. Statistical analysis has also been used to analyze the hydrogen bond structures of water–methanol mixtures; the methanol–water binary solutions showed three hydrated structures, which transformed when the molar ratios were 0.7 and 0.25.

When the microstructure of a solution changes, its macroscopic properties also change, which is an important aspect of studying the physical and chemical properties of a solution through the relationship between its microstructure and its surface tension and viscosity [22,23,24,25]. However, many connections within methanol–water mixtures remain poorly understood. In this article, we extended our work to the more important case of establishing the relationship between the microstructure and viscosity of aqueous solutions. We found that, in methanol–water mixtures, the C-O band undergoes a large shift, while the O-H band only undergoes a slight blueshift owing to the hydrogen bonds formed by the methanol and water molecules. More importantly, the structure of the hydrogen bonds affects the viscosity of the mixture. Raman spectra were used to semi-quantitatively determine the relationship between hydrogen bonding and macroscopic properties.

## 2. Results and Discussion

**Figure 1:**
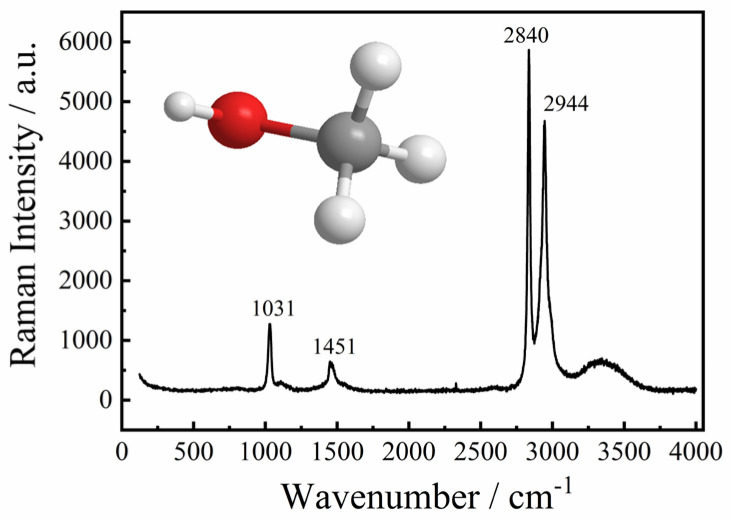
Raman spectrum of pure methanol. A schematic diagram of the monomolecular structure of methanol is shown in the upper left.

Methanol molecules are the smallest among the alcohols, consisting of one methyl group and one hydroxyl group. When methanol molecules interact with water molecules, the molecular structure is significantly affected and can be recorded by Raman spectra. Methanol molecules only contain three groups: C-O, O-H, and C-H. Changes in the O-H stretching vibration band can be used to determine hydrogen bonds, and methanol itself also has O-H bonds. Therefore, we studied the hydrogen bonding between methanol and water using the frequency shifts in the Raman peaks of methanol’s C-O and C-H bond vibrations. The frequency of the characteristic bands decreased slightly with the decrease in methanol content. The destructive effect of water on the molecular chain structure of methanol was obvious; the hydrogen bonds between methanol molecules were gradually replaced by new hydrogen bonds (CH_3_OH…H_2_O). Figure 1 shows the Raman spectrum of pure methanol. The Raman bands and their vibration modes are assigned as follows, from low to high wavenumbers: The Raman band at 1031 cm^−1^ is assigned to the C-O stretching vibration. The Raman band at 1451 cm^−1^ is assigned to the CH_3_ asymmetric deformation vibration. The Raman bands at 2840 cm^−1^ are assigned to the CH_2_ symmetrical stretching vibrations, and the Raman bands near 2944 cm^−1^ are assigned to the CH_3_ asymmetrical deformation vibrations. In the range of 3100–3800 cm^−1^, the characteristic bands correspond to OH stretching vibrations. The molecular vibration assignments of liquid methanol are shown in Table 1.

### 2.1. The Effect of Hydrogen Bonds on C-O, C-H, and O-H Bonds

**Figure 2:**
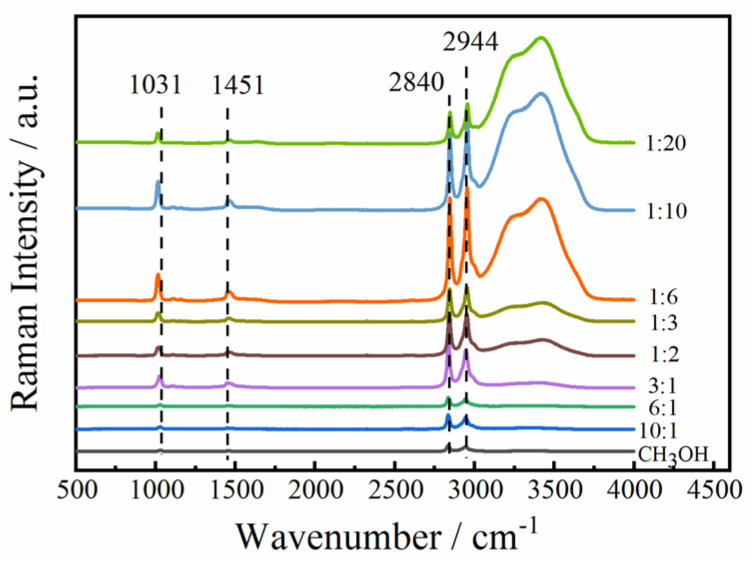
Raman spectra of pure methanol and methanol–water binary aqueous solutions (nCH_3_OH:nH_2_O) with different molar ratios.

**Figure 3:**
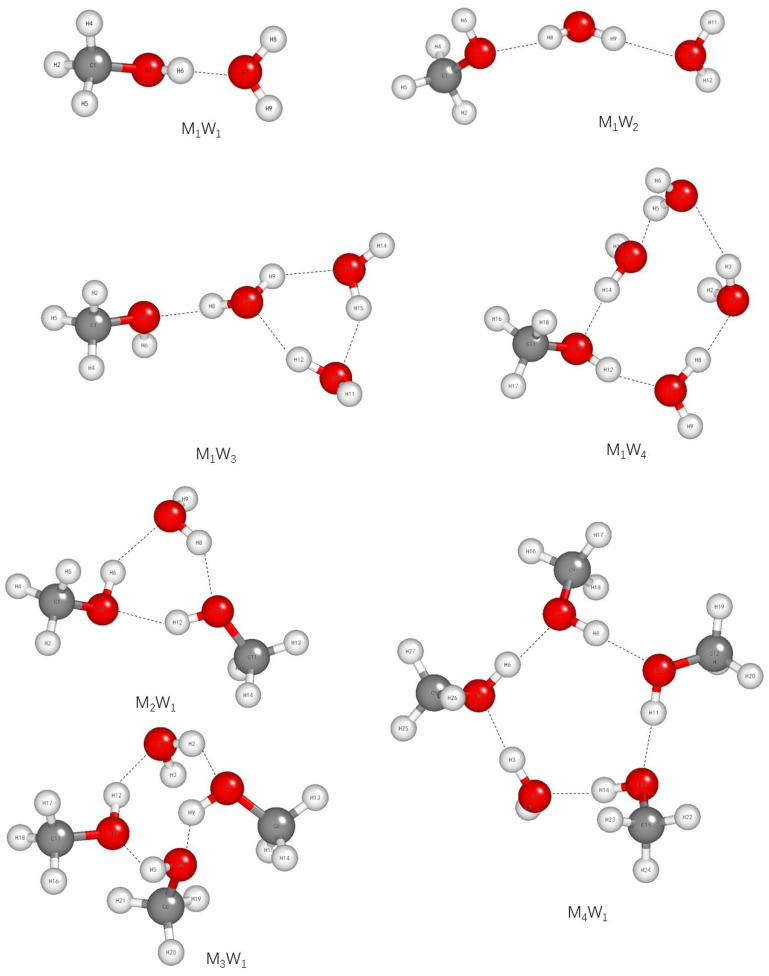
Optimized geometric structures of methanol–water clusters calculated at the B3LYP/6-311++G(d, p) level.

The Raman spectra of both neat methanol and its binary mixtures with different molar ratios are shown in Figure 2. As can be observed, two characteristic bands assigned to the C-O stretching vibration and C-H stretching vibration modes of methanol molecules in methanol–water solutions are presented. The shifts in the bands are different for all molar ratios. It is likely that the presence of a weaker band gives rise to an apparent asymmetry. This may be the result of different inter-molecular interactions and their extents in mixtures containing varying concentrations of methanol. Figure 3 presents the optimized structures of (methanol)_n_-(water)_n_ clusters (M_n_ W_n_, n = 1–4) calculated at the B3LYP/6-311++G(d, p) level using the BDF software (No. BDF-2024A).

As the methanol concentration increases, the Raman bands of the O-H stretching vibrations shift to lower wavenumbers. When the molar ratio of methanol is lower than 1:3, the characteristic band assigned to the C-O stretching vibration mode hardly shifts. The redshift of characteristic bands is assigned to the C-H stretching vibration modes. When the molar ratio of methanol is higher than 1:3, the characteristic bands assigned to the C-O stretching vibration mode are blueshifted, and the characteristic bands assigned to the C-H stretching vibration mode are redshifted. When the molar ratio of methanol is higher than 3:1, the characteristic band assigned to the C-O stretching vibration mode hardly shifts. The frequency shift with concentration in the characteristic band assigned to the C-O stretching vibration mode is shown in Figure 3; it can be inferred that the transition points of the hydrogen bond structure in the solution occur when the molar ratios of methanol to water are 1:3 and 3:1.

**Figure 4:**
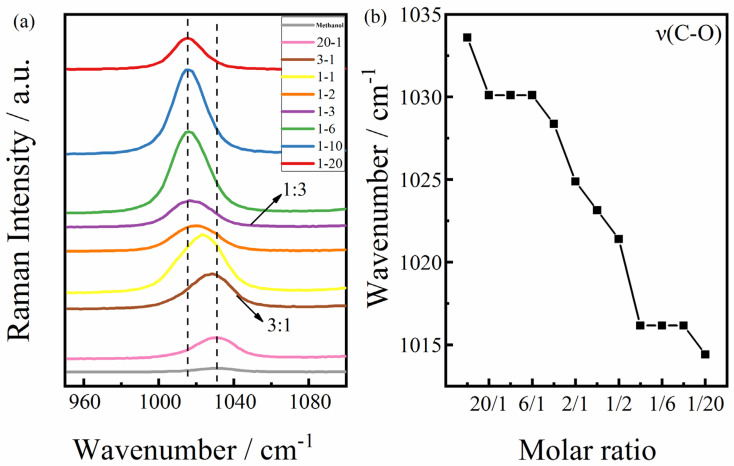
(**a**) The characteristic bands assigned to C-O stretching vibration modes in methanol aqueous solution (nCH_3_OH: nH_2_O) with different molar ratios. (**b**) Wavenumber shifts in the bands.

The hydrogen bonding between methanol molecules and water molecules in aqueous methanol solutions with varying concentrations was analyzed by measuring the Raman spectra of several groups (see Figure 4). The hydrogen bond structure formed by the interaction between methanol and water molecules was studied by the frequency shifts in the Raman bands of the C-O, O-H, and C-H bond vibrations according to the relationship between the vibrational frequency of diatomic molecules and the vibrational force constant [26]:

(1) $$\nu =\left(1/2\pi c\right)\sqrt{k/\mu }$$

where *ν* is the vibration frequency of the O-H stretching vibration mode in water molecules, *k* is the vibration force constant, *c* is the speed of light, and *μ* is the mass of a single particle. It can be observed that the vibration frequency and the vibration force constant have a proportional relationship in Equation (1). As the vibration frequency increases, the vibration force constant increases. The vibration force constant is related to the electron cloud density of the covalent bond. When the electron cloud density approaches that of any one of the two atoms, the vibration force constant decreases. Conversely, when it approaches the middle of the two atoms, the vibration force constant increases. For water molecules, the electron cloud density between O-H bonds is biased towards the oxygen atom due to its high electronegativity. The hydrogen atoms in the O-H bond interact with different groups in the methanol molecule to form hydrogen bonds. The O-H bond length in the H_2_O molecule increases, and the density of the electron cloud shifts more towards the O atom, away from the central position. Therefore, the wavenumber of the O-H stretching vibration mode of water molecules decreases with an increase in the relative content of methanol in the solution.

Wopenka et al. [27] laid the foundation for a quantitative analysis of Raman spectroscopy and simplified the expression of Raman scattering intensity to

(2) $$I=K^{\prime \prime }N\sigma I_{L}$$

where $K^{\prime \prime }$ is the scale factor, *N* is the number of molecules with Raman activity, *σ* is the Raman cross-section, and *I_L_* is the intensity of the excitation light source. It can be seen from Equation (2) that the Raman scattering intensity is directly proportional to the concentration of the measured substance. The result indicates that the concentration of the measured substance can be obtained from the intensity of the Raman characteristic band. Importantly, using the relative intensity ratio as a parameter can eliminate the influence of test conditions on the measurement results. [28,29,30,31] The expression is as follows:

(3) $$N=\frac{\sigma_{R}}{\sigma }\frac{I}{I_{R}}N_{R}$$

Thereinto, *R* is the selected reference system. $\sigma_{R}/\sigma$ can be regarded as a constant.

In the 1400–1550 cm^−1^ range, there is a character band corresponding to the C-H stretching vibration mode. The intensity of the character band is noted as I_I_. The intensity of the band that is attributed to the C-O stretching vibration mode is noted as I_H_. The C-H stretching vibration mode is used as the reference. The relative intensity is calculated as *I_H_/I_I_*. The relationship between the concentration of the methanol and the relative intensity of the character band is obtained using MATLAB R2014a.

(4) $$n=0.1126\left[I_{H}/I_{I}\right]+0.4408$$

where *n* is the concentration of the methanol.

The Raman spectra demonstrate the hydrogen bonding structure of methanol molecules and water molecules. Methanol molecules and water molecules can form different molecular networks through hydrogen bonding, so many researchers have studied cluster structures in aqueous methanol solutions through hydrogen bonding [26,28,32,33,34,35,36,37]. In summary, through a large number of theoretical studies on the effect of hydrogen bonding on the structures of methanol–water binary solutions, when the molar ratios are 0.7 and 0.25 (i.e., molar ratios = 3:1 and 1:3), the structure changes. This change was recorded by Raman spectroscopy. By observing the frequency shift in the Raman characteristic bands of the solution, it can also be inferred that the transition points of the hydrogen bond structure in the solution occur when the molar ratios of methanol and water are 1:3 and 3:1, respectively.

The structural anomalies of water, methanol, and their mixtures pose challenges for the scientific community. Changes in the hydrogen bond structures of binary aqueous solutions exhibit unique local structures. When the molar ratio of methanol is lower than 1:3, the tetrahedral structure of water is rarely destroyed by methanol. A small number of water molecules in the tetrahedral structure are replaced by methanol molecules through hydrogen bonds. When the molar ratio of methanol is 1:3, the tetrahedral structure of water is completely destroyed and more water molecules rapidly form a monocyclic structure with methanol molecules. In this study, when the molar ratio of methanol was higher than 3:1, the hydrogen bonds of the methanol–water complexes began to break and methanol–methanol complexes were formed instead. The hydrogen bond length between methanol–methanol complexes increased, resulting in a reduction in the bond length of the C-O bond. A decrease in the bond length of the C-O bond led to an increase in the corresponding vibrational force constant. According to the diatomic molecular interaction formula (Equation (1)), as the vibrational force constant increases, the frequency also increases, indicating that the Raman band of the C-O bond shifts to a higher wavenumber, which is consistent with the shifts in the characteristic bands of the Raman spectra.

### 2.2. The Effect of Hydrogen Bond Interactions on the Viscosity of the Solution

**Figure 5:**
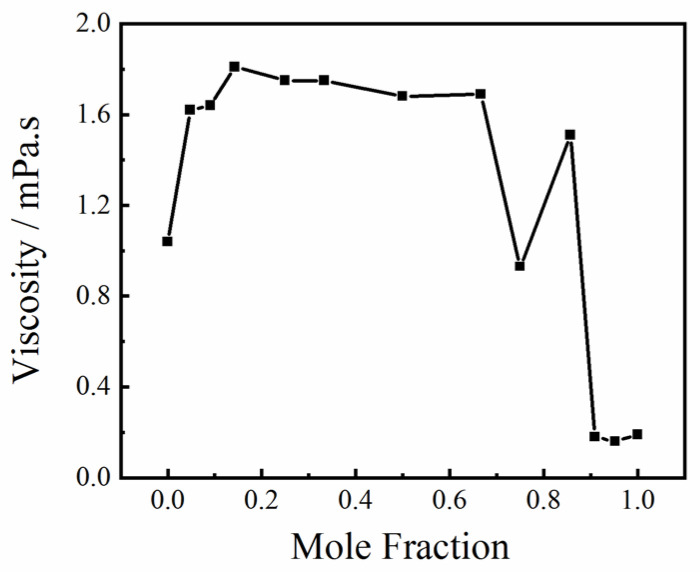
Viscosity characteristics of pure methanol and methanol–water binary mixtures with different mole fractions.

When liquid flows, the opposing forces between the layers are called viscous forces. Viscosity is an important physical quantity used to measure the magnitude of internal friction, which is affected by factors such as molecular structure and temperature. As shown in Figure 5, the viscosity of the methanol–water binary mixture shows an irregular relationship with concentration. The viscosity of the mixed solution is greater than that of the two pure solutions.

In addition to the experimental measurements, the viscosity of mixed aqueous solutions and its influencing factors were studied using correlations and predictions with a viscosity calculation model. The Eyring equation [38], Grunberg–Nissan equation [39], and Weiland equation [40] are currently the most commonly used viscosity models. Compared with the Eyring and Grunberg–Nissan equations, the Weiland equation has more advantages and a wider application range. We used Weiland’s equation to predict viscosity, as it accounts for temperature as well as mass fraction and other influencing factors. The equation is expressed as follows:

(5) $$\eta_{mix}=\frac{w_{1}}{w_{1}+w_{2}}\eta_{1}+\frac{w_{2}}{w_{1}+w_{2}}\eta_{2}$$

In the formula, $\eta_{mix}$ represents the viscosity of the mixed aqueous solution, $w_{1}$ and $w_{2}$ represent the mass fractions of methanol and pure water, respectively, and $\eta_{1}$ and $\eta_{2}$ represent the viscosity of the pure methanol and pure water, respectively. Since the solutions contained only methanol and water, we simplified the formula to the following:

(6) $$\eta_{mix}=w_{1}\eta_{1}+w_{2}\eta_{2}$$

According to the above formula, the viscosity of an aqueous methanol solution is influenced by the relative contents of the two substances in the solution. When $w_{1}\eta_{1}>>w_{2}\eta_{2}$, the viscosity of the methanol aqueous solution is infinitely close to the viscosity of methanol.

Viscosity is directly affected by the structure of the hydrogen bond network between the methanol and water molecules. With a change in the concentration of the solution, different hydrogen bond network structures are formed between the molecules in the solution, and the molecular cluster structure and viscosity both change. When a trace amount of methanol was added to pure water, the viscosity of the aqueous methanol solution increased slightly. A small amount of water molecules in the tetrahedral structure of water was replaced by methanol molecules, and the methanol and water molecules formed a tetrahedral structure through hydrogen bonding. When the molar ratio of methanol added to pure water was higher than 1:3, the viscosity varied in a small range. The tetrahedral structure was completely destroyed, and the water molecules rapidly formed a monocyclic structure with methanol molecules. When the molar ratio of methanol was higher than 3:1, the viscosity reduced quickly. The hydrogen bonds of the methanol–water complexes began to break and methanol–methanol complexes formed. This shows that the tetrahedral structure formed by methanol molecules and water molecules increases the viscosity of the solution and that the ring structure formed by methanol molecules and water molecules has little effect on the viscosity. When the methanol–methanol complex begins to form, the viscosity decreases rapidly. The influence of hydrogen bonding on the viscosity of an aqueous methanol solution is complex; the spatial structure of a hydrogen bond network, the form of hydrogen bond interactions, and the spatial directionality of hydrogen bond strength all affect viscosity.

## 3. Experimental Methods

### 3.1. Sample and Preparation

The methanol used in this experiment had over 99.5% purity and was not further purified before the experiment. Water was deionized by UPD-I-20T HYJD-D purchased from Sichuan ULUPURE Ultrapure Technology Co., Ltd. (Chengdu, China), with a resistivity of 18.25 MΩ·cm. All concentrations are expressed as molar ratios (nCH_3_OH:nH_2_O).

### 3.2. Experiment Apparatus

The Raman spectra were measured using a Renishaw inVia Qontor Raman spectrometer (Renishaw, inVia Qontor, Wotton-under-Edge, UK) equipped with a Leica microscope, a 532 nm solid-state laser, and an electron-multiplier CCD (charge-coupled device) detector. In the tests of the sample solutions, the scanning range was 0 to 4000 cm^−1^ and the resolution was ±1 cm^−1^; the exposure time was 10 s and the accumulation time was 1 s. The samples were placed in 5 mm diameter tubes and the spectra were acquired at room temperature.

The viscosities were measured by an NDJ-5S-type digital display viscometer purchased from Shanghai Precision Instrument Co., Ltd. (Shanghai, China). The measuring range was 1–100,000 m Pa·s, the accuracy was ±0.2 m Pa·s, Rotor 0 was selected, and the speed was either 30 RPM or 60 RPM.

The optimized structures of (methanol)n-(water)n clusters (M_n_ W_n_, n = 1–4) calculated at the B3LYP/6-311++G(d, p) level were determined using the BDF software.

## 4. Conclusions and Perspectives

Raman spectroscopy was used to study the microscopic hydrogen bond structure in a methanol solution. Combined with the correlation of solution viscosity with concentration, this study showed that in a pure water system, the hydrogen bond structure changed with the addition of methanol. The relative content of the two solutes in the methanol–water binary mixture is different, and the hydrogen bond network structure of the methanol and water molecules is also changed under the action of hydrogen bonds. By observing the frequency shift in the Raman characteristic bands of the solution, it can be inferred that the transition points of the hydrogen bond structure in the solution occur when the molar ratios of methanol and water are 1:3 and 3:1, respectively. When the molar ratio of the mixed solution is higher than 3:1, methanol molecules no longer form a hydrogen bond structure with water molecules and instead start to form methanol–methanol-type complexes. The change in the hydrogen bond network structure of the solution caused a great change in the viscosity of the mixed solution, indicating that the change in the solution viscosity was related to the structure of the hydrogen bond network.

## Figures and Tables

**Table 1:** Molecular vibration assignments of pure methanol.

Serial Number	Wavenumber, m^−1^	Assignments	Ref.
1	1031	C-O stretching vibration	[20,24,25]
2	1451	CH_3_ asymmetrical deformation vibration	[20,24,25]
3	2840	CH_2_ symmetrical stretching vibration	[20,24,25]
4	2944	CH_3_ asymmetrical deformation vibration	[20,24,25]
5	3100–3800	OH stretching vibration	[20,24,25]

## Data Availability

The original contributions presented in this study are included in the article. Further inquiries can be directed to the corresponding authors.
